# Loss of TANGO1 Leads to Absence of Bone Mineralization

**DOI:** 10.1002/jbm4.10451

**Published:** 2021-01-13

**Authors:** Brecht Guillemyn, Sheela Nampoothiri, Delfien Syx, Fransiska Malfait, Sofie Symoens

**Affiliations:** ^1^ Department of Biomolecular Medicine Center for Medical Genetics Ghent, Ghent University Hospital Ghent Belgium; ^2^ Department of Pediatric Genetics Amrita Institute of Medical Sciences & Research Centre Cochin India

**Keywords:** COLLAGEN SECRETION, COLLAGENOPATHY, COPII VESICLES, OSTEOGENESIS IMPERFECTA, TANGO1

## Abstract

*TANGO1* (transport and Golgi organization‐1 homolog) encodes a transmembrane protein, which is located at endoplasmic reticulum (ER) exit sites where it binds bulky cargo, such as collagens, in the lumen and recruits membranes from the ER‐Golgi intermediate compartment (ERGIC) to create an export route for cargo secretion. Mice lacking *Mia3* (murine TANGO1 orthologue) show defective secretion of numerous procollagens and lead to neonatal lethality due to insufficient bone mineralization. Recently, aberrant expression of truncated TANGO1 in humans has been shown to cause a mild‐to‐moderate severe collagenopathy associated with dentinogenesis imperfecta, short stature, skeletal abnormalities, diabetes mellitus, and mild intellectual disability. We now show for the first time that complete loss of TANGO1 results in human embryonic lethality with near‐total bone loss and phenocopies the situation of *Mia3* ^*−/−*^ mice. Whole‐exome sequencing on genomic DNA (gDNA) of an aborted fetus of Indian descent revealed a homozygous 4‐base pair (4‐bp) deletion in *TANGO1* that is heterozygously present in both healthy parents. Parental fibroblast studies showed decreased TANGO1 mRNA expression and protein levels. Type I collagen secretion and extracellular matrix organization were normal, supporting a threshold model for clinical phenotype development. As such, our report broadens the phenotypic and mutational spectrum of *TANGO1*‐related collagenopathies, and underscores the crucial role of TANGO1 for normal bone development, of which deficiency results in a severe‐to‐lethal form of osteochondrodysplasia. © 2021 American Society for Bone and Mineral Research © 2020 The Authors. *JBMR Plus* published by Wiley Periodicals LLC. on behalf of American Society for Bone and Mineral Research.

## 1 Introduction

**Fig. 1:**
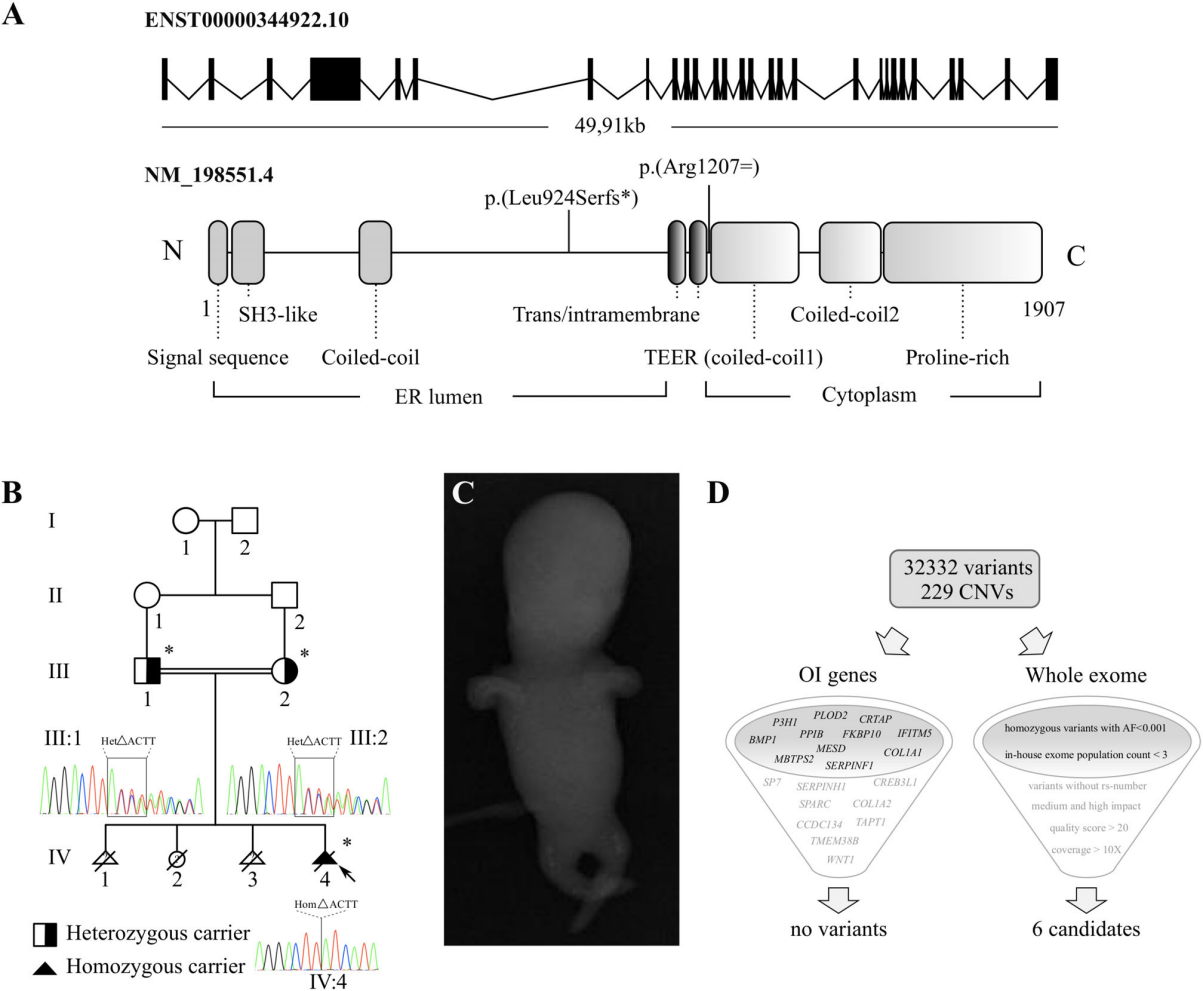
Structure of the TANGO1 gene and protein, clinical findings of the Indian consanguineous family, and overview of the exome filter strategy. (*A*) The *TANGO1* gene consists of 28 exons (8142 bp) and comprises 49.91 kb on chromosome 1. Full‐length TANGO1 protein consists of 1907 amino acids, and contains luminal, transmembrane, and cytosolic domains. TANGO1‐short lacks the luminal portion and contains the domains shown in color gradient. The earlier identified pathogenic variant p.(Arg1207=) and our variant p.(Leu924Serfs*) are highlighted. (*B*) Pedigree of the Indian family with reported consanguinity. The proband (IV:4) is indicated with an arrow, asterisks denote family members available for molecular testing, the parents are obligate carriers of the identified variant. (*C*) Anterior–posterior radiographs from IV:4 at 13 weeks of gestation reveal short and bowed extremities and a remarkable undermineralization throughout the whole body. (*D*) WES generated 32332 variants in the proband that differed from the consensus genomic sequence (GRCh37/hg19), and ExomeDepth called 229 rare CNVs (93 duplications and 136 deletions). No variant or CNV was retained when filtering the whole exome dataset for the known OI genes (left), 6 candidates resulted from retaining variants that correspond to the combination of 6 filter settings (right).

**Fig. 2:**
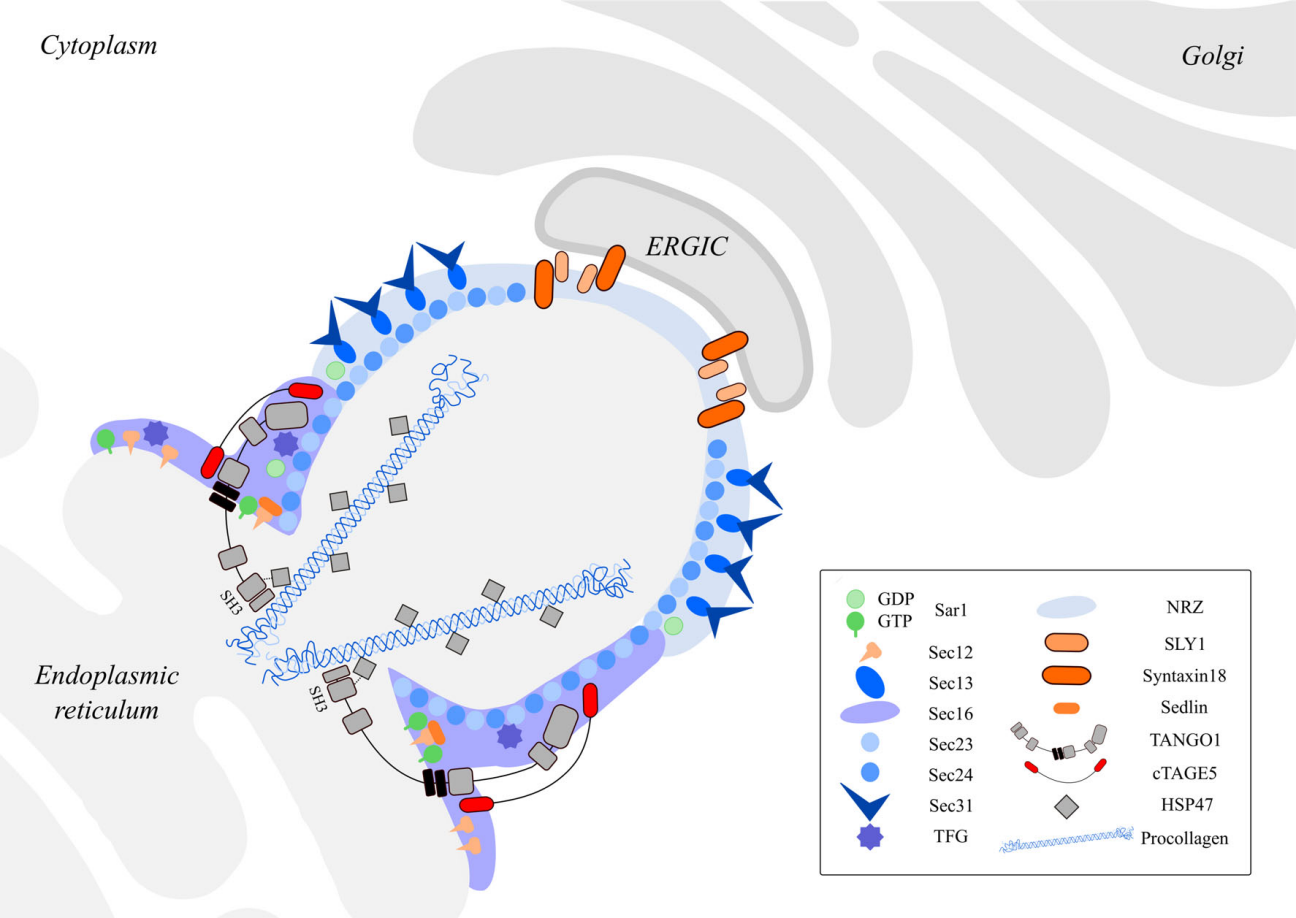
Function of TANGO1 in procollagen packaging and transport at ERES. Newly synthesized proteins exit the ER via coat protein complex II (COPII) vesicles, but procollagens form prefibrils that are too large to fit into typical COPII vesicles.^(^ ^8^, ^22^, ^23^ ^)^ cTAGE5 first binds the ER‐membrane protein Sec12 and concentrates it to the ERES. Procollagen is subsequently guided to ERES when TANGO1 binds HSP47 through its ER SH3‐like luminal domain. TANGO1 and cTAGE5 bind to the COPII inner coat components Sec23 and Sec24 and recruit the NRZ tethering complex (NBAS/RINT1/ZW10), which in turn recruits ERGIC membranes to the ERES. TANGO1/cTAGE5 finally assembles the fusion machinery SLY1 and syntaxin18 that drives incorporation of ERGIC membranes into ERES. The transmembrane helices of TANGO1 prevent the miscibility of ERGIC and ERES membranes while allowing the transfer of collagen from the lumen of the ER to the ERGIC membranes via a tunnel.^(^ ^5^, ^8^, ^22^ ^)^ Proteins are shown by symbols as defined in the figure legend, all different domains of TANGO1 are shown and the SH3‐like collagen interacting site is highlighted with a dotted line.

The identification of the transport and Golgi organization‐1 homolog (TANGO1) protein as a key player between cytoplasmic coat protein complex (COP) II coats and cargoes, kindled the interest in the pathway by which cells export bulky secretory cargoes such as collagens.^(^ ^1^, ^2^, ^3^ ^)^ Its encoding gene *TANGO1*, located at chromosome 1q41, comprises 8142 base pairs (bp) which code for two distinct alternative spliced isoforms, full‐length TANGO1 and TANGO1‐short. Full‐length TANGO1 contains an N‐terminal signal sequence, followed by a luminal SH3‐like domain, two putative transmembrane domains, coiled‐coil domains, and a cytoplasmic proline‐rich domain (PRD), respectively (Fig. 1*A*).^(^ ^1^ ^)^ TANGO1‐short lacks the luminal portion and has been shown to back up the function of full length TANGO1 in collagen export, and vice versa.^(^ ^4^ ^)^ The interaction of TANGO1 with collagens requires binding of the SH3‐like domain to the collagen‐specific chaperone HSP47,^(^ ^1^, ^5^ ^)^ whereas the PRD domain of TANGO1 interacts with endoplasmic reticulum (ER) exit sites (ERES), and the COPII components Sec23/Sec24.^(^ ^1^ ^)^ TANGO1 has also been demonstrated to bind cTAGE5, another ERES‐resident protein, which recruits Sec12 and in turn enrolls more COPII coats.^(^ ^1^, ^6^, ^7^ ^)^ As such, TANGO1 has at least three major functions: it recruits and binds COPII coats; it engages ER–Golgi intermediate compartment 53 (ERGIC‐53)–containing membranes; and it binds HSP47, which then collects procollagens.^(^ ^8^, ^9^, ^10^ ^)^ A scheme of TANGO1 functions in creating a route for procollagen export is shown in Fig. 2.

Mice lacking *Mia3* (murine TANGO1 orthologue) display a chondrodysplasia that becomes already apparent as early as 15.5 to 16.5 days post‐coitum (dpc) and causes dwarfing of the fetus with shortening of the snout and limbs, fragility of multiple tissues and the skin, and peripheral edema, all leading to perinatal lethality with complete absence of the mature ossified skeleton. Further analysis showed defective secretion of numerous collagens (including collagens I, II, III, IV, VII, and IX) from chondrocytes, fibroblasts, and endothelial and mural cells.^(^ ^11^ ^)^ In this report we present a consanguineous Indian family, in whom the proband is a fetus presenting with early lethality and almost complete absence of bone formation. Whole‐exome sequencing (WES) analysis revealed a novel homozygous frameshift variant, generating a premature termination codon and predicted complete absence of the TANGO1 protein. Herewith, we underscore the previous observation that TANGO1 plays a unique role in normal embryonal skeletogenesis and add TANGO1 as a novel gene to the group of human lethal osteochondrodysplasias.

## 2 Materials and Methods

### 2.1 Study oversight and sequencing

The study was conducted in accordance with the Declaration of Helsinki, and informed consent was obtained from the parents and the age/sex matched control individuals. WES was performed on genomic DNA (gDNA) from the proband using the Human All Exon V6 enrichment kit (Agilent Technologies, Santa Clara, CA, USA), followed by paired‐end sequencing (2 × 150 bp) on a HiSeq3000 sequencer (Illumina, San Diego, CA, USA). Variant calling and analysis were performed using our in‐house developed variant annotation and querying platform “SeqPlorer” (unpublished) and ExomeDepth^(^ ^12^ ^)^ was used to call rare copy number variants (CNVs). Variants were sorted as described in the text and confirmed by bidirectional Sanger sequencing using the BigDye® Terminator Cycle Sequencing kit and the ABI 3730XL DNA Analyzer (Applied Biosystems, Foster City, CA, USA). Nucleotide numbering reflects cDNA numbering, with +1 corresponding to the A of the ATG translation initiation codon in the *TANGO1* reference sequence, amino acid residues are numbered from the first methionine residue of the protein reference sequence (GRCh38.p13, NM_198551.4). Variant nomenclature and classification is according to the HGVS and ACMG guidelines,^(^ ^13^ ^)^ and the variant was submitted to the Mutalyzer server (https://mutalyzer.nl) (output file in the Supplementary Material).

We used the GeneMatcher server (https://genematcher.org) in order to search for other *TANGO1*‐families.

### 2.2 Cell culture

Skin biopsies from the parents and age‐sex matched controls were explanted and fibroblast cultures were grown at 37°C with 5% CO_2_ in complete DMEM (Gibco, Thermo Fisher Scientific, Waltham, MA, USA) supplemented with 10% fetal bovine serum (Biochrom AG, Berlin, Germany), 1% Penicillin/Streptomycin (Gibco), 1% non‐essential amino acids (Gibco) and 0.1% Fungizone (Gibco).

### 2.3 Quantitative real‐time PCR

Total RNA was extracted in triplicate from cultured dermal fibroblasts with the RNeasy Kit (QIAGEN, Hilden, Germany), and cDNA was synthesized with the iScript cDNA Synthesis Kit (Bio‐Rad Laboratories, Hercules, CA, USA). Primer sequences are listed in Supplementary Table S1 (Integrated DNA Technologies, Coralville, IA, USA). For each cDNA sample, RT‐qPCR reactions were prepared with the addition of SsoAdvanced™ Universal SYBR® Green Supermix (Bio‐Rad Laboratories, Hercules, CA, USA), and were subsequently run in duplicate on a Roche LightCycler 480 System (Roche Diagnostics, Mannheim, Germany). Data were analyzed with qbase+ software (version 3.0; Biogazelle, Ghent, Belgium), and expression was normalized to the reference genes *HPRT1*, *RPL13A*, and *YWHAZ*.

### 2.4 Immunoblotting

For immunoblotting of TANGO1, HSP47, SEC23A, and SEC24D, protein lysates were prepared from cultured fibroblasts using a 50mM Tris–HCL buffer (pH 7.4, 150mM NaCl, 1mM ethylenediaminetetraacetic acid, 1% Triton X‐100) with protease inhibitor cocktail (Roche, Basel, Switzerland). Cell extracts were scraped, collected and centrifuged at 18,400 g  for 15 min at 4°C, and the supernatant was subsequently subjected to SDS‐PAGE (3–8% Tris‐Acetate gels for TANGO1, SEC23A, and SEC24D, 4–12% Bis‐Tris gels for HSP47) under reducing conditions (6.25% 1M dithiothreitol) (NP0335BOX; Life Technologies Europe, Merelbeke, Belgium). Proteins were blotted to a nitrocellulose membrane (3 hours wet blotting for TANGO1; dry blotting [iBlot 2 Dry Blotting System; Thermo Fisher Scientific, Waltham, MA, USA] for HSP47, SEC23A, and SEC24D). Membranes were blocked in 5% BSA (TANGO1), 2% ECL Blocking Agent (GE Healthcare, Chicago, IL, USA) (HSP47, SEC23A, and β‐tubulin), or 5% dry milk (SEC24D), incubated overnight with primary antibodies against TANGO1 (1/1000; HPA055922; Sigma‐Aldrich, St. Louis, MO, USA), HSP47 (1/1500; ab54874; Abcam, Cambridge, UK), SEC23A (1/500; ab179811; Abcam), SEC24D (1/1500; ab191566; Abcam), or β‐tubulin (1/1500; ab6046; Abcam), and subsequently incubated with horseradish peroxidase–conjugated secondary antibody (1/1500; 7074S and 7076S; Cell Signaling Technologies, Leiden, The Netherlands). Membranes were scanned with an Amersham Imager 680 System (GE Healthcare, Chicago, IL, USA), quantitation was achieved using ImageJ software (NIH, Bethesda, MD, USA; https://imagej.nih.gov/ij/) and normalized to the amount of β‐tubulin. Graphs display data points normalized to control values.

### 2.5 Immunocytochemistry

In order to study the type I collagen extracellular matrix organization, 60,000 fibroblasts cells were seeded in eight‐well Nunc Lab‐Tek chamber slides (Thermo Fisher Scientific Scientific, Waltham, MA, USA), grown in medium (composition as described above) supplemented with 25 μg/mL ascorbic acid. After 48 hours (2 days) or 120 hours (5 days), cells were fixed with 4% (wt/vol) paraformaldehyde (Sigma‐Aldrich), permeabilized with Triton X‐100 (0.5% (vol/vol) in PBS) and blocked with bovine serum albumin (BSA; 5% (wt/vol) in PBS). Cells were next incubated with the primary type I collagen (1/100; AB758; Merck Millipore, Burlington, MA, USA) and secondary antibodies (AlexaFluor488 conjugated anti‐goat (1:1500; Molecular Probes, Life Technologies, Carlsbad, CA, USA) diluted in 2% BSA/PBS. Nuclei were counterstained with DAPI (4′‐6′‐diamidino‐2‐phenylinodole hydroxychloride; Vector Laboratories, Burlingame, CA, USA). Stained preparations were analyzed using the Axio Observer.Z1 fluorescence microscope (Carl Zeiss Microscopy, Thornwood, NY, USA). Images were captured and processed with the Zen pro software (Carl Zeiss).

### 2.6 Collagen secretion assays

The pulse‐chase secretion kinetics studies for type I collagen were performed as described.^(^ ^14^, ^15^ ^)^ In brief, fibroblasts were seeded and grown for 24 hours in medium (composition as described in section 2.2 Cell culture) supplemented with 25 μg/mL ascorbic acid, labeled for 4 hours with [^14^C]‐proline and chased with fresh medium containing 2mM cold proline. Medium and cell layer procollagens were harvested at the indicated times and pepsin‐digested. Two wells for each assay were left unlabeled to provide an accurate cell count for normalization between cell lines. Normalized samples were run on 6% SDS‐PAGE gels and visualized by autoradiography.

### 2.7 Statistical analysis

Statistical analysis was performed using GraphPad Prism 8.3.1 software (GraphPad Software, Inc., La Jolla, CA, USA). The RT‐qPCR and immunoblotting results are expressed as mean ± standard error of the mean (SE) from two or three independent experiments, respectively, and the statistical significance was determined by performing one‐way analysis of variance followed by Bonferroni's test for multiple comparisons (see figure legends).

## 3 Results

### 3.1 Clinical phenotype

We report an Indian family with third degree consanguinity (Fig. 1*B*), with a history of three induced termination‐of‐pregnancy because of clinical suspicion of lethal osteogenesis imperfecta (OI) and one full‐term pregnancy. The proband (IV:4) was a fetus who on ultrasound at 13 weeks of gestation showed tetramicromelia, increased nuchal translucency thickness of 4.7 mm, absence of the nasal bone, compressibility of the skull, significant shortening of the long bone segments, and an impressive undermineralization throughout the whole body. On X‐ray, only improperly formed bones in the region of the femur and tibia could be noticed at this point (Fig. 1*C*).

The first pregnancy of the couple was terminated at 13 weeks of gestational age. The fetus (IV:1) presented with short long bones in both upper and lower limbs, fetal hydrops, nuchal translucency of 4.1 mm, a normal amniotic fluid index, and no nasal bone. Ultrasound measurements were consistent with the clinical diagnosis of lethal OI. Patient IV:2 was a girl, born after a full‐term pregnancy, with normal ultrasound during pregnancy, which was complicated by fetal hydrops. Birth weight was 2.75 kg. She suffered from generalized edema and abdominal distension (hepatomegaly). She started vomiting after 1 month of age and had clay‐colored stools (starting at 1.5 months old). She passed away at 3 months of age, e causa ignota (presumed biliary atresia). No abnormal bone phenotype for this child was reported. The third pregnancy was also terminated at 13 weeks of gestation after ultrasound measurements of the fetus (IV:3), which revealed an increased nuchal translucency thickness of 4.6 mm and fetal hydrops, but no obvious skeletal abnormalities were seen. Both father (III:1) and mother (III:2) have normal karyotyping patterns and do not present with any clinical sign of OI. Other family members were not available for clinical assessment.

### 3.2 WES

We performed WES for the proband (IV:4) and analyzed the dataset using an in‐house–developed platform and the ExomeDepth copy number variant (CNV) calling algorithm.^(^ ^12^ ^)^ In view of the clinical history of this family, we first analyzed the WES dataset for the presence of (likely) pathogenic variants or CNVs in known OI genes (*BMP1*, *CCDC134*, *COL1A1*, *COL1A2*, *CREB3L1*, *CRTAP*, *FKBP10*, *IFITM5*, *MESD*, *MBTPS2*, *P3H1*, *PLOD2*, *PPIB*, *SERPINF1*, *SERPINH1*, *SP7*, *SPARC*, *TAPT1*, *TMEM38B*, and *WNT1*) (Fig. 1*D*). No (likely) pathogenic variants or CNVs were detected. Subsequently, the WES dataset was analyzed applying the following filter settings: (i) homozygous variants (in view of the reported consanguinity within this family) with a medium and high impact, (ii) variants with an allele frequency of 0.001 or less (for the Ensembl Variant Effect Predictor^(^ ^16^ ^)^ and other population databases including Genome Aggregation Database [gnomAD]); (iii) variants that are present less than 3 times in our in‐house exome population; (iv) variants with a quality score of 20 or higher; (v) variants that were covered more than 10×; and (vi) variants that were not linked to an rs‐number. This resulted in six candidate variants (Fig. 1 *D*, Table 1). Literature searches, combined with the MARRVEL tool, prompted us to reject the variants in *MCRIP1*, *MROH8*, *NUTM2G*, *MUC19*, and *KRTAP9‐1* as reasonable candidates. *MCRIP1* encodes Mapk‐regulated corepressor‐interacting protein 1, which is involved in the regulation of epithelial‐mesenchymal transition, and is not yet linked to human disease. Genomewide association studies (GWASs) previously linked *MROH8* (Maestro heat‐like repeat‐containing protein family member 8) to Alzheimer's disease, whereas the exact function of *NUTM2G* is not known. *MUC19*, which encodes Mucin‐19, is linked with inflammatory response, hematopoietic progenitor and salivary gland cell differentiation through mice studies, and is associated with TN Polyagglutination Syndrome and colorectal cancer. *KRTPAP9‐1* (Keratin‐associated protein 9‐1) is a known matrix protein which functions in keratinization (Table 1). The most interesting candidate variant was a homozygous out‐of‐frame 4‐bp deletion (c.2770_2773del, p.(Leu924Serfs*)) in exon 4 of the *TANGO1* gene, which was confirmed by Sanger sequencing. Segregation analysis showed that both parents are heterozygote carriers of the *TANGO1* variant (Fig. 1*B*). In order to identify additional cases harboring *TANGO1*‐variants, we submitted this gene to the GeneMatcher server. Unfortunately, no additional case could be found. Interestingly, previously described *Mia3*‐null mice showed neonatal lethality due to severely compromised chondrocyte maturation and bone mineralization, leading to almost complete lack of bone formation.^(^ ^11^ ^)^ The striking similarity in absence of bone formation in both the *Mia3*‐null mice and the fetus described in this study and the importance of TANGO1 in collagen trafficking prompted us to perform further functional studies.

**Table 1:** Candidate Variants/Genes From Exome Sorting

Chromosomal location	Gene	Protein	Coding change	Protein change	MARRVEL‐other associations
Chromosome 1: 222,629,984–222,629,985	*TANGO1*	Transport and Golgi organization protein 1 homolog (TANGO1)	c.2770_2773del	p.(Leu924Serfs*)	Protein transport; chondrocyte development, collagen fibril organization and positive regulation of bone mineralization (*M. musculus*)
Chromosome 17: 81,823,317–81,823,318	*MCRIP1*	Mapk‐regulated corepressor‐ interacting protein 1	c.323del	p.(Pro108Leufs*)	Regulation of epithelial‐mesenchymal transition
Chromosome 20: 37,179,387–37,179,388	*MROH8*	Maestro heat‐like repeat‐containing protein family member 8	c.93_93 + 1insAGTGCCGGC CGCGGGGCCCTGTCTAT AAG	p.(His32Serfs*)	Exact function of *MROH8* is not known, but GWAS associated this gene with Alzheimer's disease^(^ ^24^ ^)^
Chromosome 9: 96,931,892–96,931,893	*NUTM2G*	NUT family member 2G	c.187C>T	p.(Pro63Ser)	Exact function of *NUTM2G* is not known
Chromosome 12: 40,490,439–40,490,440	*MUC19*	Mucin‐19	c.17500_17501insCATCAGC TGCTGTGACTGGGTCAGC TGGACTATCAGCTGGGGT GACAGGGACAACTGGAC	p.(Gly5833_Gln5834ins ProSerAlaAlaValThrGly SerAlaGlyLeuSerAlaGly ValThrGlyThrThrGly)	TN polyagglutination syndrome, colorectal cancer; inflammatory response, hematopoietic progenitor and salivary gland cell differentiation (*M. musculus*)
Chromosome 17: 41,190,343–41,190,344	*KRTAP9‐1*	Keratin‐associated protein 9‐1	c.483_484insAGCTGTGGGT CCAGCTGCTGCCAGCCT	p.(Pro161_Cys162ins SerCysGlySerSerCysCys GlnPro)	Keratinization

SeqPlorer and MARRVEL (http://www.marrvel.org/) were used to sort the variants from most likely pathogenic (top) to probably pathogenic (bottom) and to investigate the functional annotation of these genes and rare variants, respectively. The variant in the *TANGO1* gene is listed as the best candidate.MARRVEL = Model Organism Aggregated Resources for Rare Variant Exploration.

### 3.3 Fibroblast studies

**Fig. 3:**
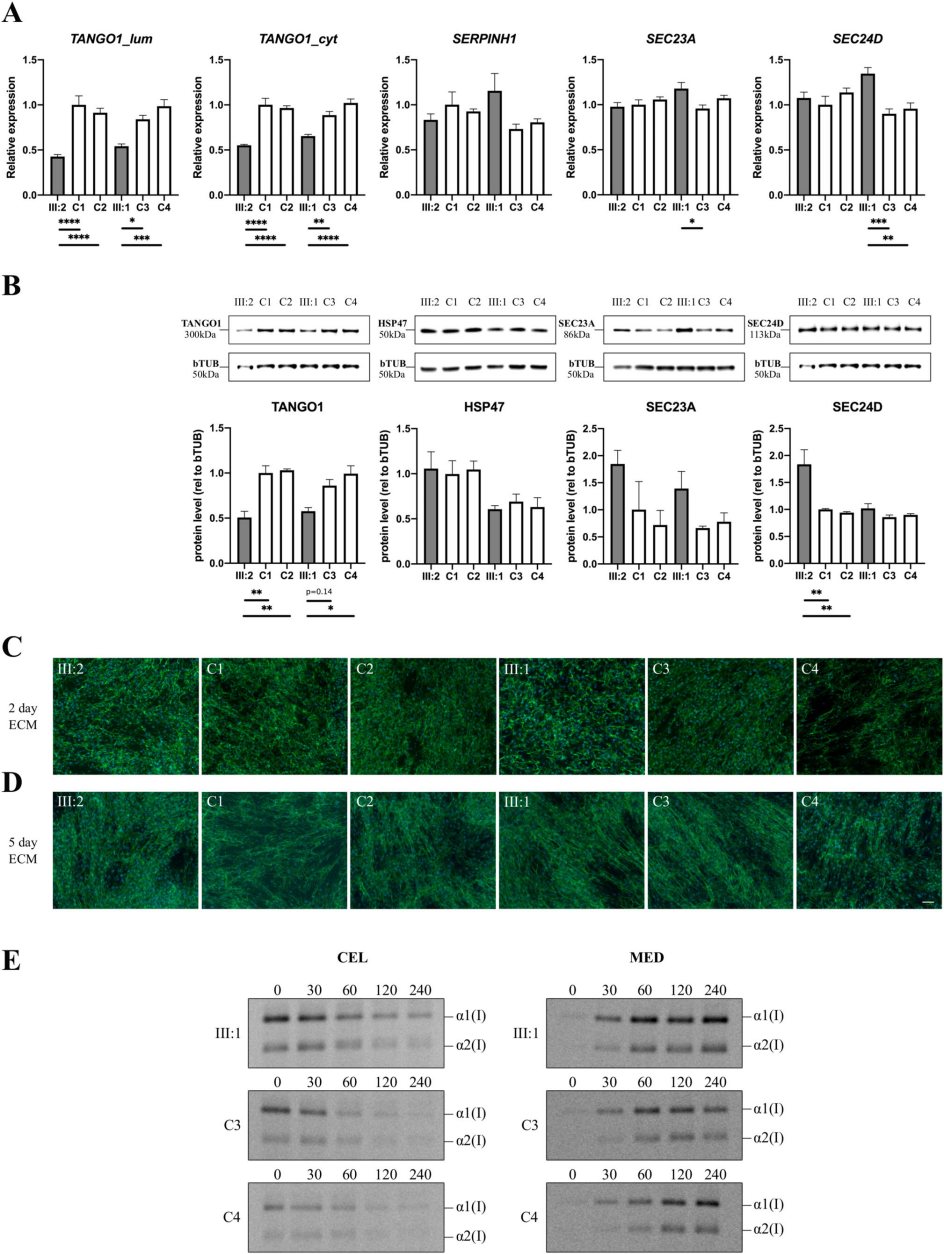
Variant characterization in fibroblasts of heterozygous carriers, compared to age/sex matched control individuals. (*A*) RT‐qPCR expression analysis for *TANGO1_lum*, *TANGO1_cyt*, *SERPINH1*, *SEC23A*, and *SEC24D*. Data are expressed as mean ± SE, each graph represents the average quantification of three experiments performed in duplicate (6 data points). (*B*) Western blot analysis for TANGO1 (full length), HSP47, SEC23A, and SEC24D. Data are expressed as mean ± SE, each graph represents the average quantification for each experiment. (*C*,*D*) Immunocytochemical analysis for extracellular matrix formation of type I collagen after 2 and 5 days of ascorbic acid stimulation. Nuclei are counterstained with DAPI. Scale bar = 50 μm. (*E*) Pulse‐chase secretion kinetics of the intracellular (CEL, left panels) and secreted (MED, right panels) type I collagen chains during a 240‐min time range. C = control; CEL = intracellular; MED = secreted.

Because no fibroblasts were available from the proband, functional studies were conducted on parental fibroblasts in order to gain insights into the pathophysiology underlying the *TANGO1* variant. The identified frameshift variant is located in the luminal N‐terminal part of the gene (Fig. 1*A*). Introduction of a premature termination codon is predicted to lead to nonsense‐mediated decay (NMD), causing loss of both the luminal and cytosolic part of the protein and thus loss of expression of the full‐length TANGO1. To investigate this, we first designed specific RT‐qPCR assays to measure mRNA expression levels for both the luminal (*TANGO1_lum*) and the cytosolic (*TANGO1_cyt*) proportion, respectively. Expression levels of *TANGO1_cyt* and *TANGO1_lum* in both parental RNA samples (III:1 and III:2) were halved when compared to age/sex matched controls (C1, C2, C3, and C4) (Fig. 3*A*), pointing toward unstable mutant mRNA molecules for both luminal and cytosolic portions of TANGO1 and thus loss of expression of the mutant allele. Immunoblotting with an antibody against the full length TANGO1 protein (recognizing amino acids 422–509, located in the luminal part upstream of the identified frameshift), revealed that full‐length TANGO1 protein levels were reduced to about 50% in both heterozygous parents (III:1 and III:2), thereby confirming NMD of the mutant *TANGO1* allele (Fig. 3*B*).

Next, we investigated whether decreased TANGO1 levels could affect the normal functioning of TANGO1 in collagen secretion. Therefore, we first focused on key components in TANGO1 trafficking at ERES: the “upstream” HSP47 and “downstream” SEC23A/SEC24D components (Fig. 2). The collagen‐specific chaperone HSP47 (encoded by *SERPINH1*) was previously shown to bind to the SH3‐like domain of TANGO1.^(^ ^5^ ^)^ Both transcriptional (RT‐qPCR) and translational (immunoblotting) levels of *SERPINH1* (Fig. 3*A*,*B*) were normal in heterozygous carriers. However, the downstream targets *SEC23A* and *SEC24D* tended to be (slightly) upregulated at both mRNA and protein levels (Fig. 3*A*,*B*).

To investigate a possible effect on large cargo trafficking, we assessed collagen organization and secretion. First, immunocytochemistry for type I collagen, after 2 or 5 days of ascorbic acid stimulation, revealed that type I collagen extracellular matrix organization was normal in the heterozygous parents (Fig. 3*C*,*D*). In addition, a pulse‐chase secretion assay showed normal secretion kinetics for type I collagen in the heterozygous parents compared to age/sex matched control individuals (Fig. 3*E*).

## 4 Discussion

In 2011, Wilson and colleagues^(^ ^11^ ^)^ demonstrated that mice deficient for *Mia3* present with a chondrodysplasia characterized by severe dwarfism and neonatal lethality, which was caused by impaired chondrocyte maturation and lack of bone mineralization. The delay in mineralization in *Mia3* ^*−/−*^ pups was already apparent as early as 13.5 dpc, with an almost complete lack of calcium deposits at 15.5 dpc when normal primary mineralization is initiated. In wild‐type mice, primary ossification is accompanied by invasion of osteoblasts, necessary for secondary mineralization, and is characterized by expression of matrix metalloproteinase 9 (Mmp9), which primes the mineralized matrix for vascular invasion. However, in mutant *Mia3* ^*−/−*^ pups, the expression of the latter was strongly reduced. Furthermore, Wilson and colleagues^(^ ^11^ ^)^ also demonstrated that in the dwarfed *Mia3* ^*−/−*^ mutants at 14.5 dpc no osteopontin‐positive osteoblasts were present, leading to absence of osteoblast‐derived type I collagen and insulin‐like growth factor‐binding protein (Igfbp6) in the perichondrium, eventually resulting in absence of ossified tissue at 18.5 dpc and thus also absence of secondary mineralization. These findings were also corroborated by histological analyses revealing defects in chondrocytic ECM deposition, absence of mineralized bone collar and well‐defined trabeculae in *Mia3* ^*−/−*^ pups. Although major chondrocyte maturation signaling axes, such as the *Indian hedgehog* and *patched* pathway, were preserved, studies on primary mouse embryonic fibroblasts revealed that timely expression of type II and type X collagen is dependent on Mia3, as is the case for the transit and maturation of several other collagens. Thereby, Wilson and colleagues^(^ ^11^ ^)^ elegantly demonstrated that the generalized dwarfing of the skeleton of the *Mia3* deficient pups is primarily driven by: the delay and arrest of chondrogenic maturation; lack of vascular recruitment; and the failure to elaborate a primary ossification center, highlighting the sensitivity of the chondrogenic/skeletogenic processes to defects in the secretory pathway.

Over the past decade, and in addition to these murine studies, in vitro exploration in which HeLa^(^ ^1^, ^4^, ^10^ ^)^ and RDEB/FB/C7 cells^(^ ^17^ ^)^ were knocked out for TANGO1 (using siRNAs and clustered regularly interspaced short palindromic repeats [CRISPR] technology) have additionally demonstrated a crucial role of TANGO1 in the context of cargo loading at ERES and collagen secretion.

Recently, Lekszas and colleagues^(^ ^18^ ^)^ identified a homozygous synonymous substitution (c.3621A>G, p.(Arg1207=)) in *TANGO1*, leading to skipping of exon 8 and the generation of a truncated TANGO1 protein, which is dispersed in the ER and impairs type I collagen secretion. Affected patients of this family presented with dentinogenesis imperfecta in both primary and permanent dentitions and/or a delayed eruption of the permanent teeth, various skeletal abnormalities including growth retardation with proportionate short stature, platyspondyly, scoliosis, osteopenia, brachydactyly and clinodactyly, primary obesity, insulin‐dependent diabetes mellitus, sensorineural hearing loss, and mild intellectual disability.^(^ ^18^, ^19^ ^)^ Some of the patients also presented with mild retinopathy, early onset periodontitis with premature tooth loss, hydronephrosis, and microalbuminuria. No overt bone phenotype was described in the heterozygous carriers of this family.

In this study, we describe an Indian family in which the proband (fetus IV:4) presented with early embryonic lethality due to almost complete lack of bone formation. The fetus is homozygous for an out‐of‐frame 4‐bp deletion in *TANGO1*. Analogous to the previously described family,^(^ ^18^ ^)^ both heterozygous parents of the family described in this study are healthy and show no overt signs of skeletal involvement. We show that in the heterozygous parents both transcriptional and translational levels of TANGO1 are halved due to the identified *TANGO1* variant (Fig. 3*A*,*B*). However, this decrease in TANGO1 does not result in obvious alterations in type I collagen secretion and organization in the extracellular matrix, and thus further corroborates the earlier hypothesis that TANGO1 haploinsufficiency does not lead to a discernible clinical phenotype, similar to *Mia3* ^+/−^ mice which do not show an overt phenotype.^(^ ^18^ ^)^ Nevertheless, the decreased expression of TANGO1 due to the presence of the heterozygous 4‐bp deletion variant allows to deduce that homozygosity for this variant would result in complete deficiency of the TANGO1 protein and result in human embryonic lethality, thereby phenocopying the *Mia3*‐null mice.^(^ ^11^ ^)^ Interestingly, although secretion of different collagens, including collagens type I, II, III, IV, and IX, was disrupted in *Mia3*‐null mice, collagen fibrils were still detected in the ECM, but they were more dispersed and diminished in number.^(^ ^11^ ^)^ As such, our and previous data^(^ ^4^, ^11^ ^)^ point toward a compensational mechanism, whereby secretion of collagens (and other bulky cargos) is still possible, because the wild‐type allele(s) of (both) full‐length and the short isoform of TANGO1 and/or another ERES resident protein compensate for the loss of expression of the mutant allele. Notwithstanding, it remains a conundrum why a trend toward increased RNA and protein levels of the COPII components SEC23A and SEC24D is detected in the fibroblasts of the parents (Fig. 3*A*,*B*). A possible explanation is that, similar to TANGO1‐short which backs up the function of full length TANGO1 in collagen export,^(^ ^4^ ^)^ cTAGE5 can compensate for this loss, as it has been shown to be a coreceptor of TANGO1 at ERES.^(^ ^6^ ^)^

An interesting point of discussion is the difference in severity of the phenotype presented in patients carrying the homozygous synonymous substitution (c.3621A>G, p.(Arg1207=)) (first family, mild‐to‐moderate severe phenotype)^(^ ^18^, ^19^ ^)^ versus the homozygous out‐of‐frame 4‐bp deletion (c.2770_2773del, p.(Leu924Serfs*)) described in this report. The homozygous synonymous variant causes skipping of exon 8 in most products, thereby affecting the beginning of the cytoplasmic portion of TANGO1 (Fig. 1*A*, downstream of the luminal portion), and resulting in decreased collagen secretion.^(^ ^18^ ^)^ In contrast, the homozygous out‐of‐frame deletion described here affects the luminal portion of TANGO1, upstream of the earlier reported synonymous variant, and mimics a full‐null situation as is seen in *Mia3* ^−/−^ pups.^(^ ^11^ ^)^ Our findings thereby further underscore and add to the previously postulated threshold model, where the disease manifests in a “mild(er)” form when the ratio of truncated versus normal protein exceeds a critical level^(^ ^18^ ^)^, and a disease severity that is not viable in cases where no functional TANGO1 is present.

In conclusion, we report the first homozygous frameshift mutation in *TANGO1*, predicted to result in complete loss of TANGO1 in a human fetus with an embryonic lethal osteochondrodysplasia. Similar to *Mia3* knockout mice, the affected fetus shows a near‐complete absence of bone formation, further suggesting a key role for TANGO1 in early human embryonic development, especially in the first stages of bone formation. A more complete understanding of the role of *TANGO1* within the secretory pathway and its link to diabetes will have to come from the identification of additional patients with defects in *TANGO1*, and also by including this gene in prenatal exome strategies.^(^ ^20^, ^21^ ^)^

## Disclosures

DS is a postdoctoral fellow of the Research Foundation Flanders (12Q5920N). FM is a clinical investigator of the Research Foundation Flanders (1842318N). All authors state that they have no conflicts of interest to disclose.

## Author Contributions

**Brecht Guillemyn:** Conceptualization; data curation; formal analysis; investigation; methodology; validation; writing‐original draft; writing‐review and editing. **Sheela Nampoothiri:** Data curation; investigation; resources; writing‐review and editing. **Delfien Syx:** Conceptualization; writing‐review and editing. **Fransiska Malfait:** Conceptualization; supervision; writing‐original draft; writing‐review and editing. **Sofie Symoens:** Conceptualization; formal analysis; investigation; methodology; supervision; writing‐original draft; writing‐review and editing.

5

### Peer Review

The peer review history for this article is available at https://publons.com/publon/10.1002/jbm4.10451.

## Supporting information


**Supplementary Table S1.** Primers for RT‐qPCR
