# Supplementary material for: Loss of TANGO1 Leads to Absence of Bone Mineralization
Source: JBMR Plus. 2021 Jan 13;5(3):e10451. doi: 10.1002/jbm4.10451 (PMC7990155; doi:10.1002/jbm4.10451)

**SUPPLEMENTAL DATA**

|  | **Antisense** | **Sense** |
| --- | --- | --- |
| *TANGO1_lum* | ctaaggaaagggaacctgaac | gtggtgcttctgagttgtaa |
| *TANGO1_cyt* | taccaccacccattcgata | gcctggtgcaaattctct |
| *SERPINH1* | gctgcaaatcgtggagat | ttcagctgctctttggttag |
| *SEC23A* | gcacatgtcagtggaagata | cattatgctgattgacaacctc |
| *SEC24D* | tcatcaagccctttggc | tctgactggtccactctc |
| *HPRT1* | tgacactggcaaaacaatgca | ggtccttttcaccagcaagct |
| *YWHAZ* | acttttggtacattgtggcttcaa | ccgccaggacaaaccagtat |
| *RLP13A* | gagcaaggaaagggtcttag | actggttgctcttcctattg |
|  |  |  |

**Table S1. Primers for RT-qPCR**

**Output Mutalyzer**


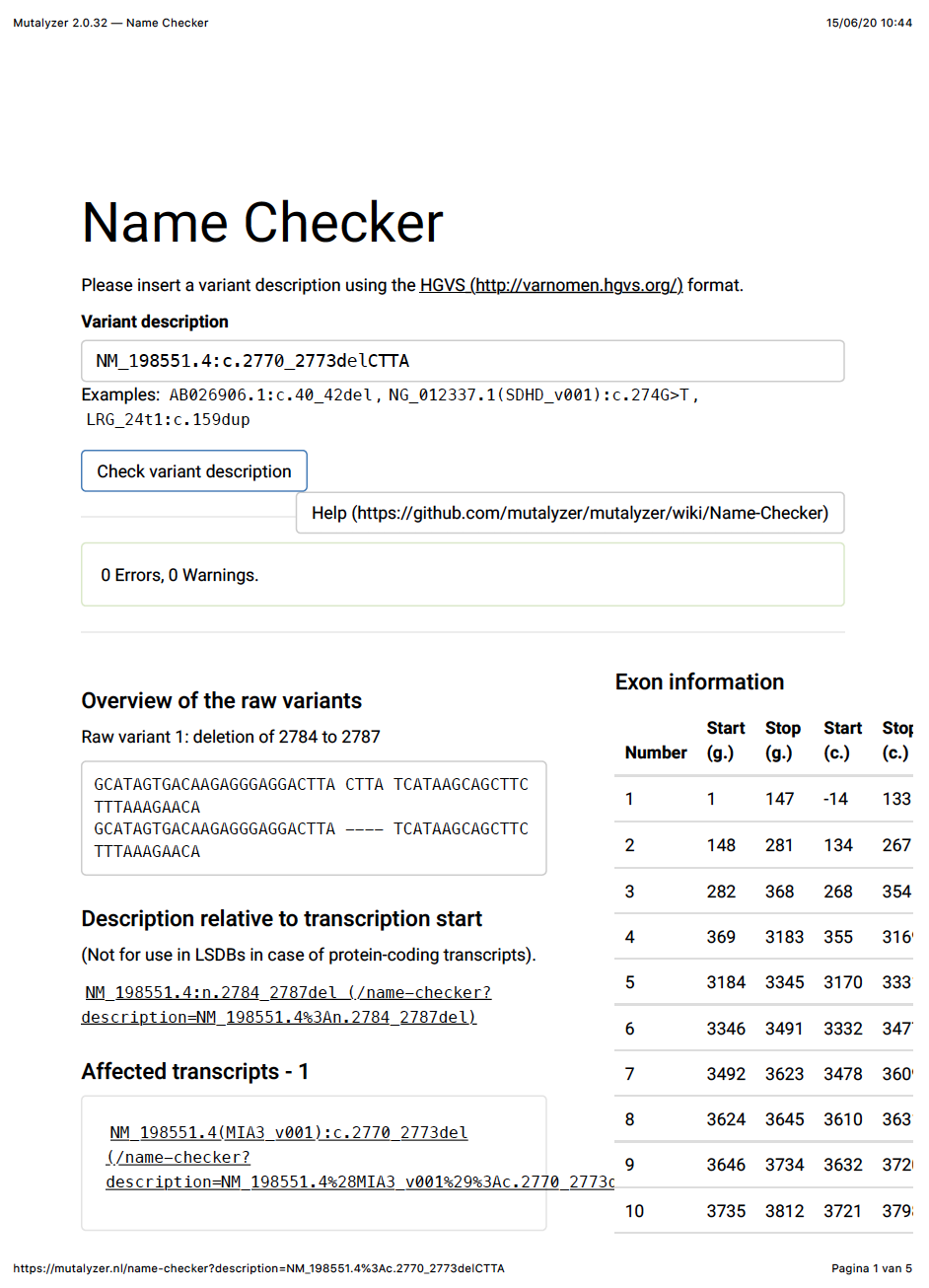


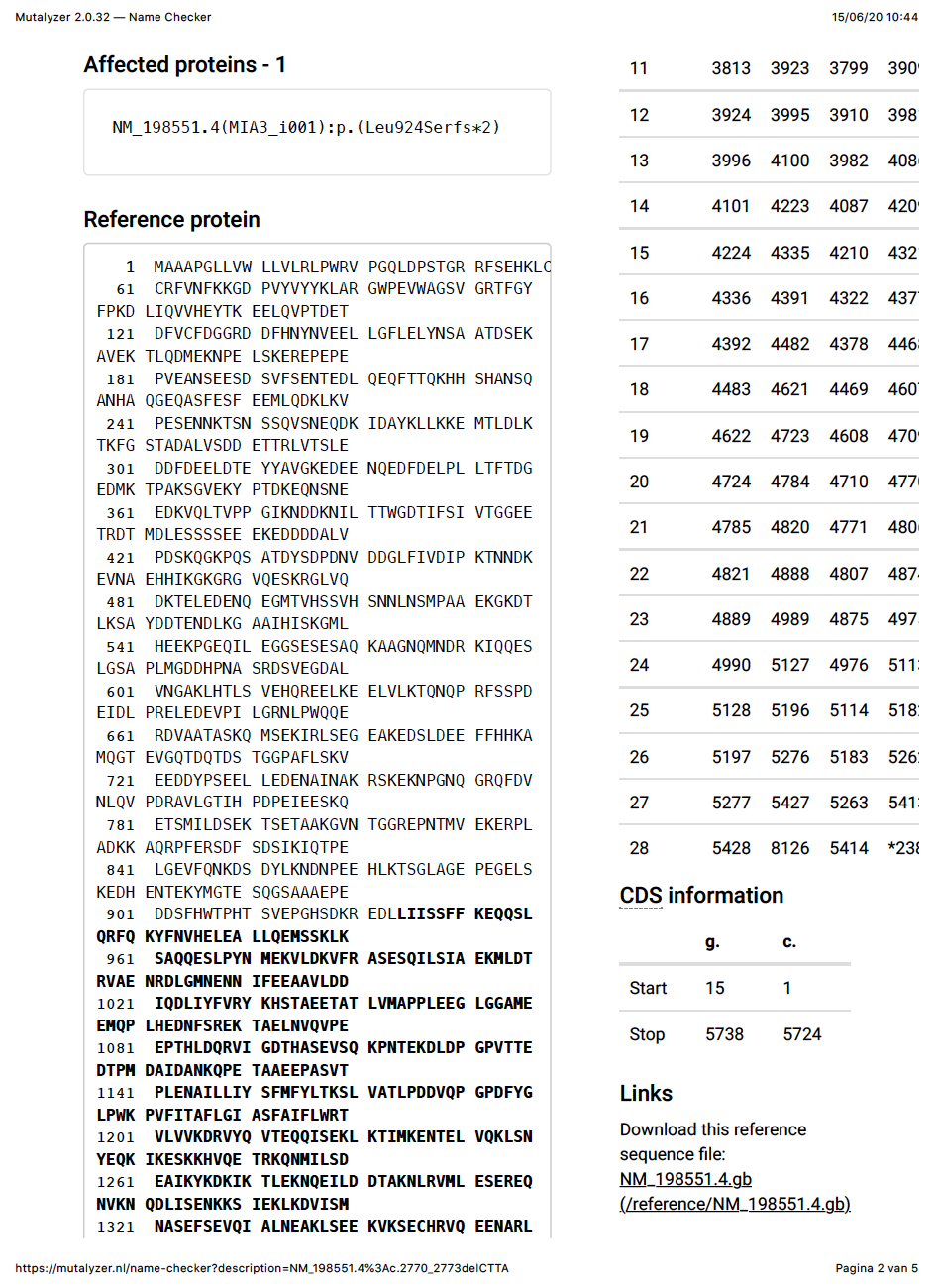


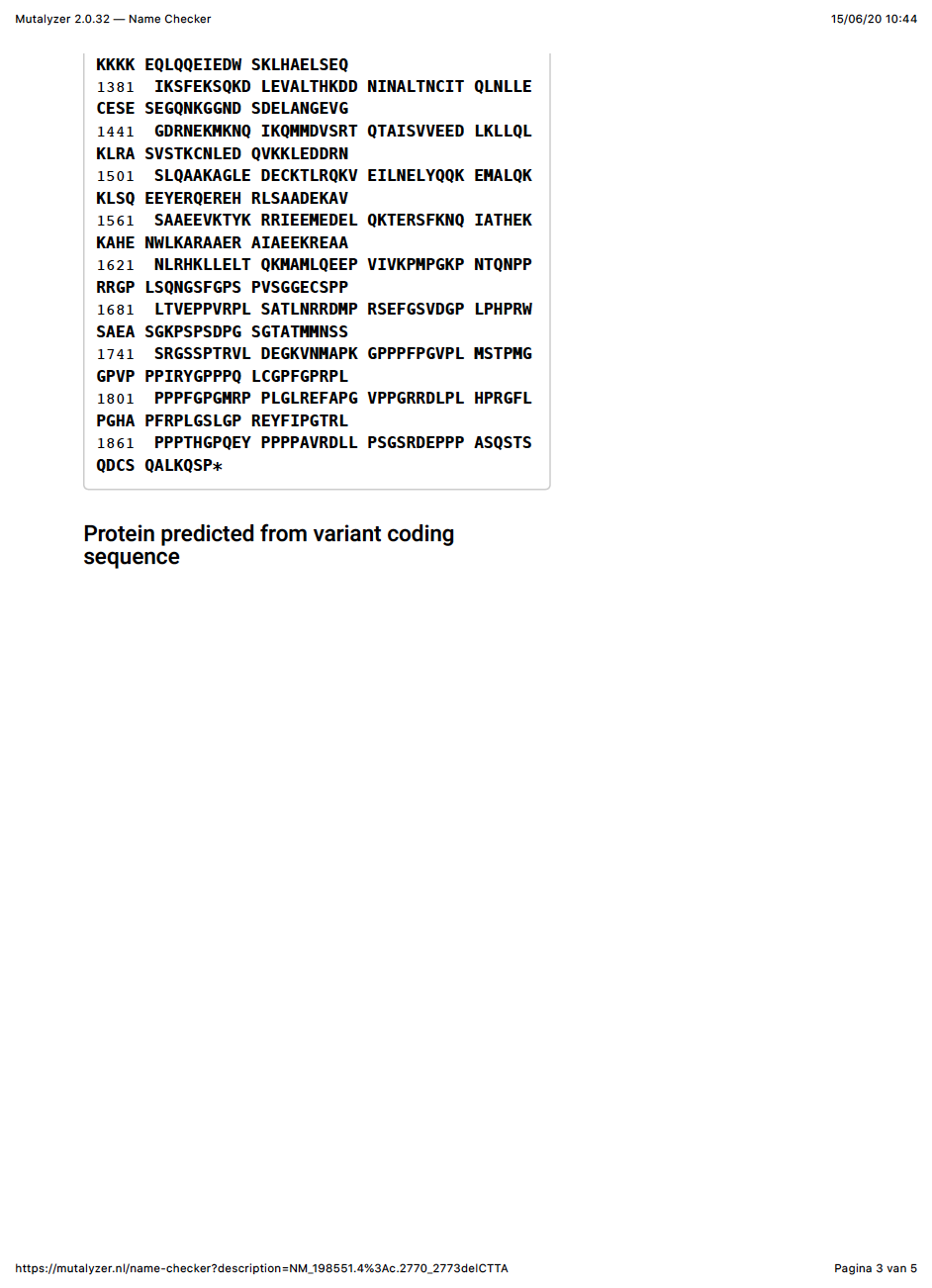


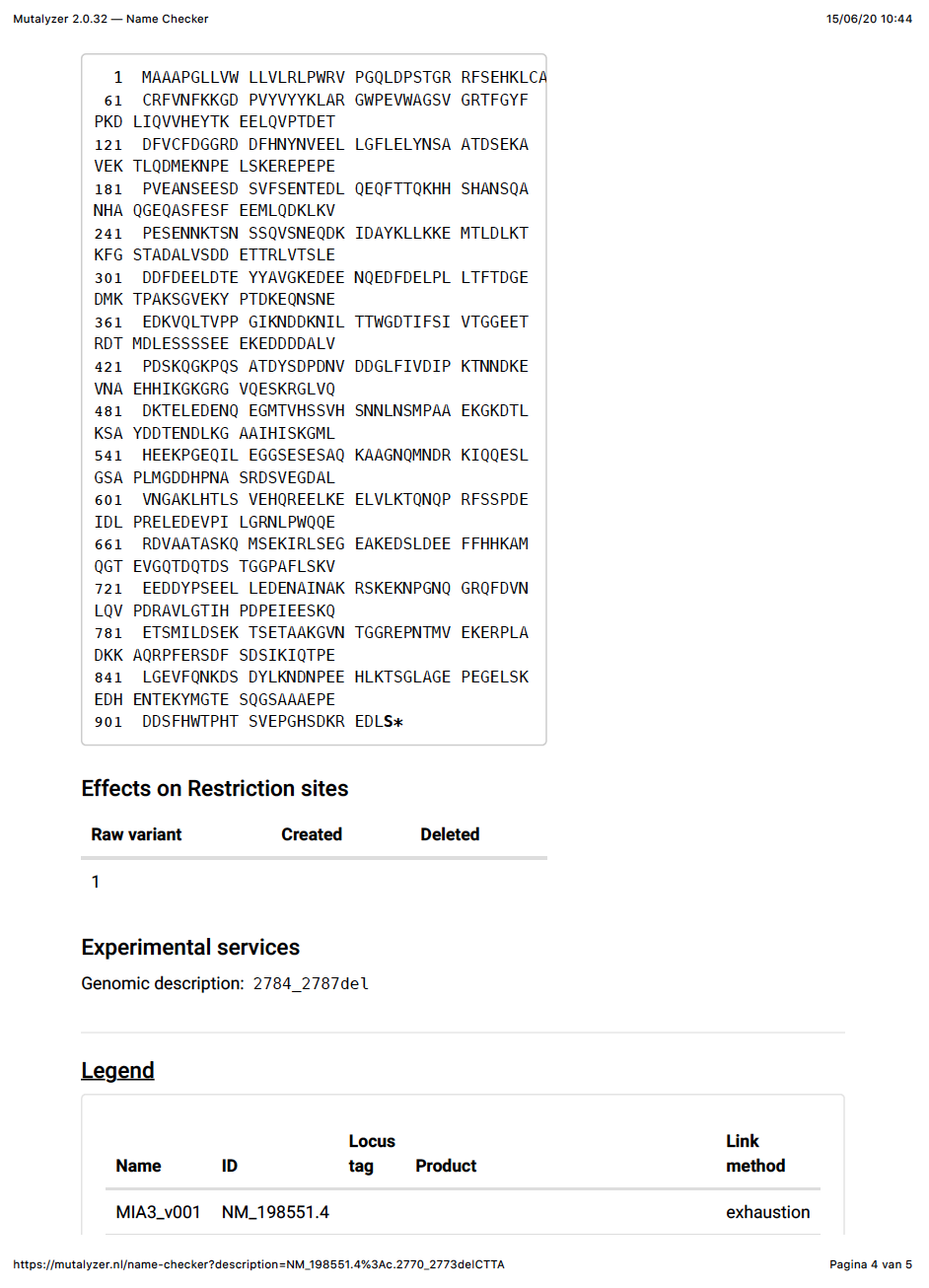


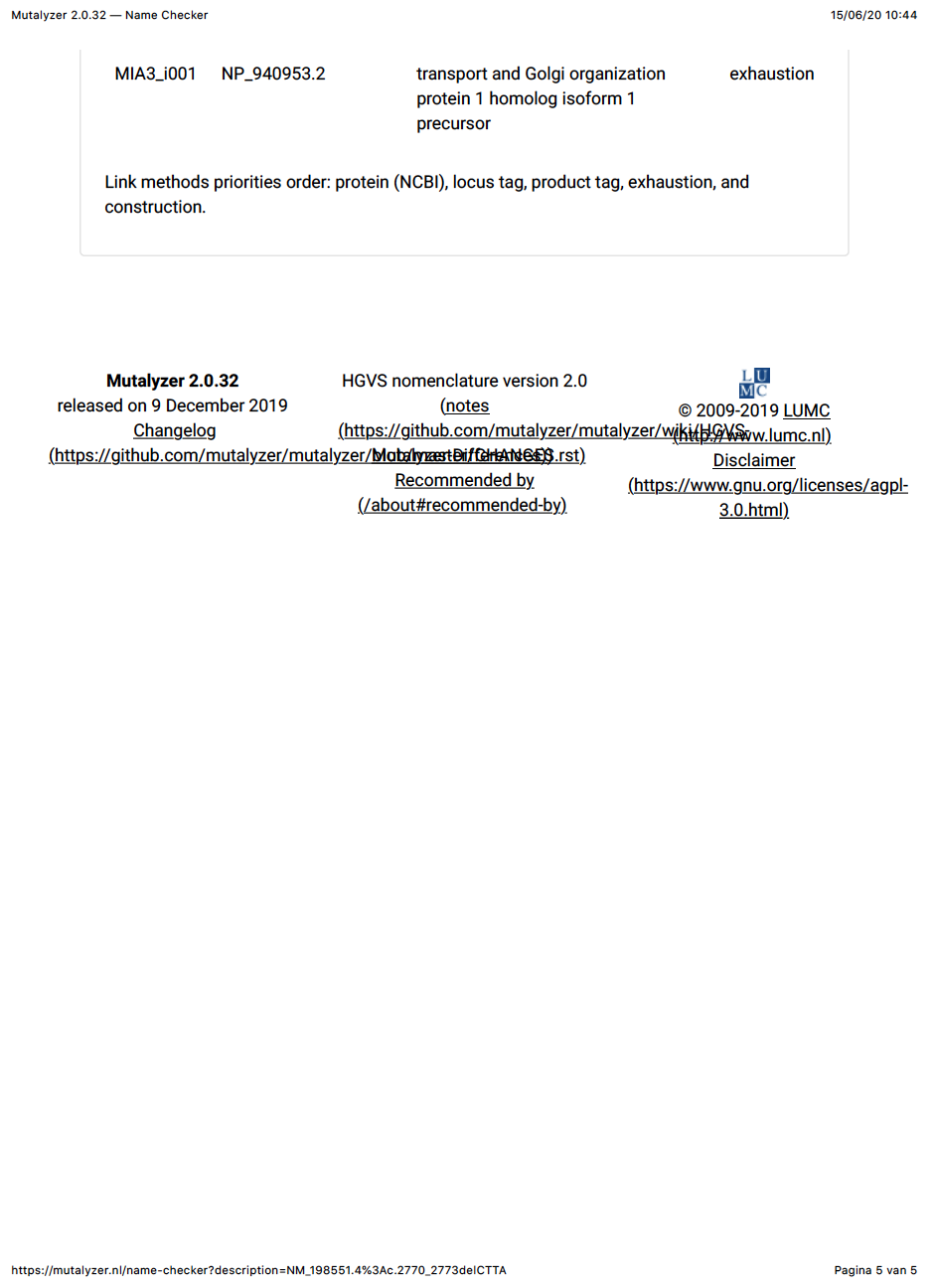

Supplement: Supplementary file 1 — Supplementary Table S1. Primers for RT‐qPCR [file JBM4-5-e10451-s001.docx]
